# Theory of Mind Development in Children With Congenital Visual Impairment: Role of Visual Impairment and Verbal Ability

**DOI:** 10.1002/pchj.70063

**Published:** 2025-11-14

**Authors:** Yong Yang, Boyao Zhao, Linli Xie, Buxin Han

**Affiliations:** ^1^ CAS Key Laboratory of Mental Health, Institute of Psychology Chinese Academy of Sciences Beijing China; ^2^ Department of Psychology University of Chinese Academy of Sciences Beijing China

**Keywords:** blindness, cognition, compensation, congenital visual impairment, language, theory of mind

## Abstract

This study aims to explore the theory of mind (ToM) status in individuals with congenital visual impairment (CVI) and identify key predictive factors. For Study 1, the false‐belief task was used to assess ToM ability in children aged 7–10 years (60 with normal sight, 33 with legal blindness, and 23 with total blindness). The results showed that children with total blindness had significantly lower false‐belief scores than sighted children, with those with legal blindness performing in between. In the first‐order false‐belief task, verbal ability only moderated differences between children with total blindness and sighted children. Meanwhile, in the second‐order false‐belief task, verbal ability moderated differences between children with total blindness and sighted children and between children with legal blindness and sighted children. For Study 2, the faux pas task was used to examine the roles of age, residual vision, and verbal ability in ToM development among 166 adolescents aged 7–19 years with CVI. While age and verbal ability significantly predicted ToM development, residual vision had no significant predictive effect. In conclusion, compared with sighted children, those with CVI show delayed ToM development, though children with legal blindness perform better than those with total blindness. Age and verbal ability are key predictors of ToM development in children with CVI. Thus, in the early stages of ToM development, maximizing the use of residual vision and other senses is crucial. Further, enhancing verbal abilities, such as through using mental state terms in conversations and reading literary works, can mitigate the negative impact of CVI. Finally, intervention strategies should be tailored to age characteristics.

## 1 Introduction

Theory of mind (ToM), a crucial component of the human mind, refers to an individual's ability to understand and attribute mental states to themselves and others (Premack and Woodruff 1978; Quesque and Rossetti 2020). ToM develops throughout infancy, adolescence, and adulthood (Mahy et al. 2014). Moreover, it is an important component of social cognition (Le et al. 2024). In this context, embodied cognition, the view that the perceptual system plays a crucial role in cognitive processes, explains human cognitive behavior (Farina 2021), and language plays a vital role in ToM development (Meinhardt‐Injac et al. 2020; Osterhaus and Koerber 2021; Siu and Cheung 2022). Given this background, blindness provides a unique perspective for understanding the origins of the human mind. Specifically, vision is the dominant modality through which infants learn about the world before language acquisition.

Thus, for children with congenital visual impairment (CVI), language becomes the most important means of understanding their environment. Clarifying the development of ToM in children with CVI helps clarify the origins of the human mind and inform evidence‐based interventions. However, the respective roles of visual impairment and language in ToM development in children and adolescents with CVI require further investigation.

ToM fosters empathy, reduces misunderstandings, and enhances self‐awareness by enabling individuals to understand their own and others' mental states. Consequently, children and adolescents with CVI may face greater challenges in mental health and interpersonal relationships than sighted peers. Better understanding the factors promoting ToM development among this population would provide essential evidence for targeted interventions aiming to improve mental health, interpersonal functioning, social integration, and personal growth.

### 1.1 Theory of Embodied Cognition and Impact of Visual Impairment

According to the theory of embodied cognition (TEC), the body underlies representation and cognition, influencing the acquisition and use of knowledge (Barsalou 2008; Farina 2021). As visual signals are the primary source of information about the social world, visual impairment influences representation and cognition. First, visual impairment leads to deficits in body representation (Schott et al. 2021) and changes in sensory representation (Bedny and Saxe 2012). Early visual impairment also impacts motor representation relating to others' body movements (Imbiriba et al. 2013). Notably, these changes in the representational system are likely to further affect one's ability to interpret others' mental states.

Second, research on perspective taking, a crucial component of ToM, in adults with blindness has demonstrated that they exhibit shorter late positive potential (LPP) latencies at frontal leads and longer LPP latencies at right temporoparietal leads compared with sighted controls (Papageorgiou et al. 2020). The temporoparietal junction and medial prefrontal cortex are key brain regions for ToM development (Schurz et al. 2014). Blind children perform poorly in visuospatial perspective taking compared with sighted children (Miletic 1995). Understanding false belief (the ability to recognize that someone can hold a mistaken belief) engages mental rotation (Xie et al. 2018). Children with blindness have poor mental rotation ability compared with sighted children (Koustriava and Papadopoulos 2010), suggesting that ToM of individuals with CVI may also be affected.

Finally, people with blindness have drastically different sensory experiences than those with normal sight. While audition plays a compensatory role in their social assessment, it does not replace the role of vision (Sorokowska et al. 2018). For infants with severe visual impairment, joint attention must be established through tactile, auditory, and olfactory cues (Colus and de Souza 2019). Children with CVI, especially those with severe impairments, are unable to experience joint visual attention, which is a critical component of social understanding, potentially preventing them from interpreting others' mental states through shared visual experiences and contributing to delayed ToM development (Minter et al. 1998).

The human mind is represented within a multidimensional psychological space, encompassing the representation of entire cognitive systems (Conway et al. 2019). Therefore, this study postulates that CVI impedes ToM development and uses TEC to explore ToM development in children with CVI.

### 1.2 ToM in Children With CVI

Few empirical studies have investigated ToM development in children with CVI, and knowledge in this area remains limited, with a consensus yet to be achieved. Additionally, studies generally involve very small samples of children with CVI (Bartoli et al. 2019; Brambring and Asbrock 2010; Green et al. 2004; Mcalpine and Moore 1995; Minter et al. 1998; Roch‐Levecq 2006). For example, Mcalpine and Moore (1995) recruited 15 children with blindness, identifying a delay in the first‐order false beliefs among children with blindness for the first time. Subsequently, Minter et al. (1998), Green et al. (2004), and Roch‐Levecq (2006) generally reported similar findings. These studies matched the age and verbal ability of control groups and found that children with blindness who were visually impaired since birth had delayed development of first‐order false beliefs. In two studies, the children with visual impairment were totally blind or displayed minimal light perception (Green et al. 2004; Minter et al. 1998). Additionally, Peterson et al. (2000), Green et al. (2004), and Brambring and Asbrock (2010) found that the delay in the development of first‐order false beliefs was delayed by approximately 4–7 years for children with blindness compared with sighted children.

However, two studies that also matched age and verbal ability found that children with CVI exhibited similar performance on false belief tasks compared with sighted children (Bartoli et al. 2019; Pijnacker et al. 2012). Nevertheless, these studies did not define CVI: it is possible that the children in these studies lost sight after the onset of the critical period for first‐order false belief development (3 years; Wellman et al. 2001). Additionally, these studies included a higher number of children with low vision than those with total blindness: Pijnacker et al. (2012) recruited 21 children with low vision (visual acuity: 0.04–0.3; mean: 0.11) and three children with total blindness, and Bartoli et al. (2019) recruited nine and eight children with low vision and total blindness, respectively. According to Roch‐Levecq (2006), the visual acuity of children with visual impairment is significantly correlated with their scores in false belief tasks.

Besides unclear definitions of CVI and children with residual vision, the inconsistent results may be explained by the etiology of blindness. For many children, the cause of blindness is related to traumatic brain injuries or premature birth, which influence ToM development (Lin et al. 2021; Witt et al. 2018). However, while some studies failed to report the etiology (Green et al. 2004; Mcalpine and Moore 1995; Peterson et al. 2000), others failed to appropriately control for it (Bartoli et al. 2019; Minter et al. 1998; Pijnacker et al. 2012; Roch‐Levecq 2006).

Verbal ability plays a vital role in ToM development, and correlations have been found between verbal ability and ToM for children with blindness (Bartoli et al. 2019; Green et al. 2004). Notably, however, previous studies have focused on the key role of verbal ability in the ToM development of sighted people. Verbal ability plays a compensatory role for older people who are outperformed in ToM by young people (Mahy et al. 2014). Longitudinal studies have demonstrated that ToM development in sighted children was predicted by their verbal ability (Osterhaus and Koerber 2021; Siu and Cheung 2022). Children's verbal ability mediates the relationship between age and ToM (Bigelow et al. 2021). Between adolescence and young adulthood, the increase in children's ability to recognize faux pas aligns with improvements in their verbal ability (Meinhardt‐Injac et al. 2020). Moreover, research on children with cochlear implants has revealed that verbal ability and age impact ToM development (Aslier et al. 2020).

In previous studies of ToM development in children with CVI, the false belief task was employed as the only study paradigm. However, children with normal sight experience two sensitive periods for ToM development: the development of first‐ and second‐order false beliefs at approximately ages 3 (Wellman et al. 2001) and 5–6 years (Miller 2009), respectively. Notably, 70% of 12‐year‐olds with visual impairment could pass the false belief task (Peterson et al. 2000). The high success rate suggests a potential saturation point, particularly in older children, limiting its ability to capture subtle differences. In comparison, the faux pas task designed for older or more capable participants (Baron‐Cohen et al. 1999), provides a more nuanced measure. To address this gap in previous studies, this study employed both tasks to ensure a broader assessment of children's ToM abilities and a comprehensive understanding of ToM development in children with CVI, especially those who may have surpassed basic false‐belief understanding.

### 1.3 The Current Study

Based on previous studies and the above discussion, this study applied an inclusion criterion for participants and a clear definition of CVI. Blind children with traumatic brain injuries, premature birth, and autism (Begeer et al. 2014; Le et al. 2024) were excluded from the study. Moreover, the sense of sight has a critical period for development during the first 3 years of life, similar to ToM (Bao et al. 2017). Therefore, we defined CVI as visual impairment occurring at or before the age of 3 years.

The loss of sensory input in one modality can lead to perceptual deficits and compensation. Research on the perspective‐taking skills of adults with blindness has demonstrated that loss and compensation coexist (Papageorgiou et al. 2020). Blind people rely heavily on verbal ability in their daily lives; however, whether verbal ability moderates ToM development in children with CVI remains unclear. Study 1, which uses a cross‐sectional design, hypothesizes that CVI is associated with ToM development in children with CVI. Moreover, it hypothesizes that verbal ability plays a moderating role in this association, constructing a moderating model to test the interaction effect of visual impairment and verbal ability on ToM.

Although visual impairment is linked to delayed ToM development, ToM brain regions in sighted and congenitally blind adults are similarly localized and functionally specific (Bedny et al. 2009). Bedny and Saxe (2012) argued that a lack of visual input is not necessary for the development of social brain regions. Rather, understanding others' minds emerges through prelinguistic and linguistic phases. ToM continues to develop with increasing age and improvements in verbal ability. However, the roles of age, verbal ability, and residual vision in ToM development in children with CVI during childhood and adolescence remain unclear.

To bridge this research gap, this study constructed a regression model with a cross‐sectional design to test relationships among age, verbal ability, residual vision, and ToM in Study 2. Study 2 hypothesizes that (1) age predicts ToM development, (2) verbal ability predicts ToM development after controlling for age and residual vision, and (3) residual vision does not predict ToM development after controlling for age and verbal ability.

## 2 Study 1

### 2.1 Method

#### 2.1.1 Inclusion Criteria for the Age of Participants

Given the critical period for first‐ and second‐order false belief development and delay time, we recruited participants to control for the age at which they passed the false belief tasks, replicating previous findings and verifying the first hypothesis. According to the critical period for the first‐ and second‐order false belief of sighted children (3 and 5–6 years, respectively), we recruited sighted children aged 3–10 years old. Additionally, previous studies have demonstrated that the delay time of first‐order false belief development in children with CVI is 4–7 years (Green et al. 2004; Mcalpine and Moore 1995; Michael et al., 2010; Minter et al. 1998; Peterson et al. 2000; Roch‐Levecq 2006). Therefore, we recruited school‐aged children with CVI aged 7–14 years.

To control for residual vision of children with CVI on ToM development, we classified children with CVI into two groups based on the visual acuity at the onset of visual impairment: children with total blindness and those with legal blindness. Children with total blindness had no vision or minimal light perception at the onset of visual impairment, whereas the visual acuity of children with legal blindness ranged from greater than light perception to less than 0.3 at the onset of visual impairment.

#### 2.1.2 Participants

To examine the moderating role of verbal ability, we constructed a moderating model. As no prior studies have directly examined this interaction, we referred to evidence on the association between verbal ability and false belief tasks (for a meta‐analysis, see Milligan et al. 2007) and assumed a medium effect size of *f* ^2^ = 0.15 (Cohen 1988). With a statistical power of 1 − *β* = 0.8 and α = 0.05, we employed the pwr package in R to analyze the required sample size, determining that a sample size of at least 98 would be required. Following previous studies, we established the following exclusion criteria for participants: etiology of blindness due to brain trauma, a brain tumor, encephalitis, or premature birth; multiple disabilities; and a history of medical, neurological, or psychological problems. Accordingly, we adopted cluster sampling and recruited children with CVI from five schools for the visually impaired. Based on the age and sex of children with CVI, we recruited sighted children from a mainstream school and a preschool.

A total of 117 children with CVI (including children with total and legal blindness aged 7–14 years) were recruited in Study 1 to verify the first hypothesis and determine the age at which participants achieved the maximum score on the false belief tasks. Specifically, the CVI sample, aged 7–14 years, comprised 45 children with total blindness (boys 27; mean age 10.53 years; SD 2.18) and 72 children with legal blindness (boys 53; mean age 10.82 years; SD 2.18). Additionally, the control group, aged 3–10 years, included 124 children with normal sight (boys 71; mean age 6.69 years; SD 2.06). In total, Study 1 included 241 participants.

#### 2.1.3 Procedure

The participants were screened to exclude those with multiple disabilities to rule out the possibility of participants with autism. To ensure effective screening, before participation, psychology teachers at the school for the visually impaired who held a bachelor's or master's degree in psychology used the original Childhood Autism Rating Scale to assess the children for symptoms of autism (Schopler et al. 1980). The participants' scores were below 30, indicating no autism. Informed consent was obtained from the teachers or parents of the participants and verbal consent was obtained from all participants.

The participants were tested individually in a quiet room. They completed false belief tasks and the verbal subtests of the Chinese version of the Wechsler Intelligence Scale for Children (Zhang 2009). Upon completion of Study 1, the participants received compensation for their time and effort. The parents were interviewed via phone to confirm etiology, time of onset, and disability rating.

### 2.2 Measures

#### 2.2.1 Theory of Mind

We used the first‐ and second‐order false belief tasks to assess the participants' ToM ability. The administration procedure was similar to that in previous studies on ToM among children with blindness (Green et al. 2004; Minter et al. 1998; Peterson et al. 2000). The participants were informed that they should listen to a story and answer test (false belief/justification) and control questions. If the control questions were answered incorrectly, the child's responses were excluded from the analysis. A correct answer was assigned a value of 1, and an incorrect answer was assigned a value of 0.

*First‐order false belief task*. The participants completed two first‐order false belief tasks, each of which involved a short story: the unexpected location task and unexpected content task (Jeffrey et al., Jeffrey Farrar et al. 2017). In the unexpected content task, the researchers used a chocolate box. Peterson et al. (2000) argued that using familiar household items as deceptive containers does not significantly reduce the difficulty of false‐belief tasks for children with blindness. To the best of our knowledge, however, children with CVI could not perceive the chocolate box through tactile experience. Based on the teapot task (Minter et al. 1998) and the container task (a Coke can containing sugar and a Smarties tube that contains pencils; Green et al. 2004) and the principle of the construction of the alternative first‐order false belief task in studies on ToM in children with blindness (Brambring and Asbrock 2010), we replaced the chocolate box with an opaque thermos cup containing sunflower seeds for the following reasons:

First, these cups are household items that children with CVI would be familiar with through their tactile experience. Second, children with CVI are aware that the unexpected content is a deception when they find that the cup contains sunflower seeds because they frequently use these objects and know their function. Finally, children with CVI and sighted children had equal prior knowledge about the object being a cup. However, they were unaware that the cup contained sunflower seeds.

We invited a few children of the same age range to recognize the objects, who provided correct responses. In this way, we ensured that the participants were familiar with the items prior to the study. All participants correctly identified the items.

*Second‐order false belief task*. The participants completed two second‐order false belief tasks, each of which comprised a short story involving the change of location of an object (Marschark et al. 2019). Considering the influence of Chinese culture, we replaced the postcard in one story with a card.

#### 2.2.2 Verbal Ability

The participants' verbal ability was assessed through the vocabulary subscale of the Wechsler Intelligence Scale for Children. The participants were instructed to listen to and explain each word read aloud to them. The score for each word was 0–2.

#### 2.2.3 Statistical Analysis

In Study 1, we performed descriptive statistics, correlation analysis, and t‐tests on the main variables. Additionally, we developed a moderation model to examine the effects of verbal ability and visual impairment on the participants' false belief scores. To facilitate comparison and interpretation, all continuous variables were standardized using *z* score transformation. All analyses were conducted in SPSS.

### 2.3 Results

In Study 1, we confirmed the age at which the participants passed all false belief tasks and tested differences in the understanding of false belief among children with CVI and sighted children as well as the interaction effects of visual impairment and verbal ability.

#### 2.3.1 Age Confirmation Standards

**FIGURE 1:**
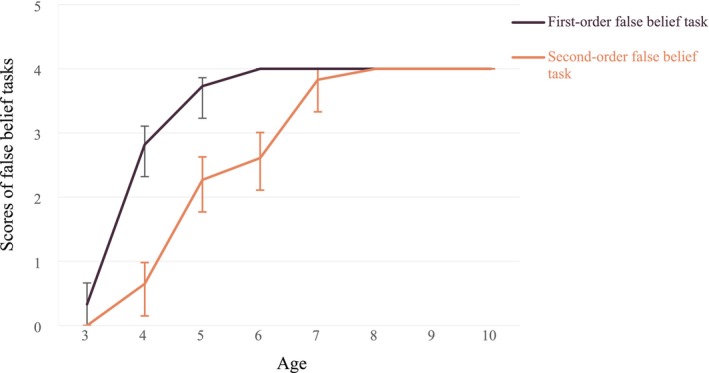
First‐ and second‐order development trajectories of the children with normal sight.

Figure 1 indicates that the first‐ and second‐order false beliefs of children with normal sight reached the full score of 4 by the age of eight.

**FIGURE 2:**
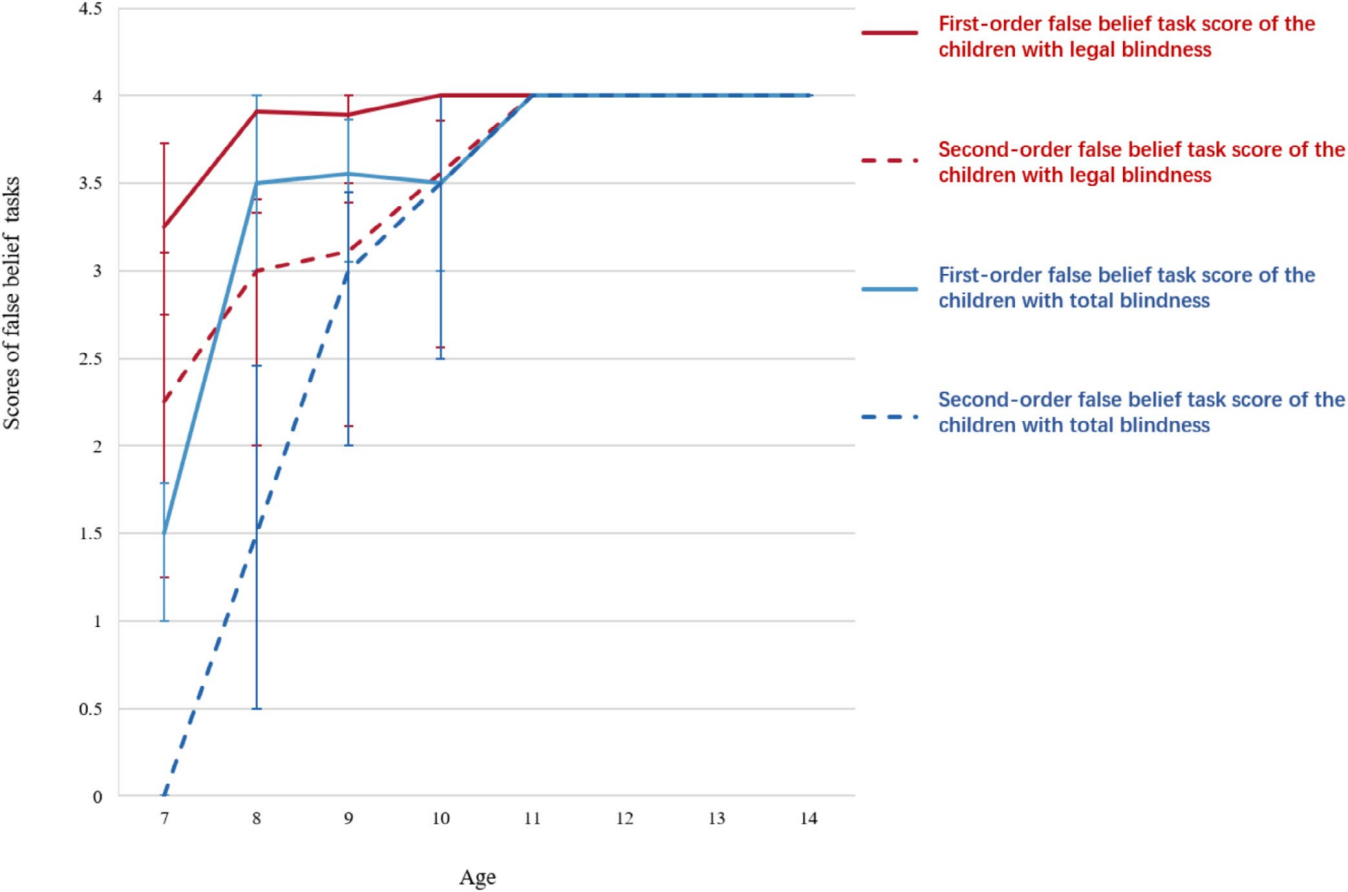
First‐ and second‐order development trajectories of the children with legal and total blindness.

Meanwhile, Figure 2 indicates that the first‐ and second‐order false beliefs of children with legal blindness and total blindness reached the full score of 4 by the age of 11 years. Thus, we selected and analyzed the data of children aged 7–10 years.

In addition, Table 1 provides a detailed overview of the sample distribution across different age groups for children with normal sight, legal blindness, and total blindness. For each age group, the table presents the number of participants and the means and standard deviations of verbal ability, first‐order false belief, and second‐order false belief.

**TABLE 1:** Descriptive statistics of key variables by age group and children with normal sight, legal blindness, and total blindness.

Age period	*n*	Verbal ability		First‐order false belief		Second‐order false belief	
		*M*	SD	*M*	SD	*M*	SD
Children with normal sight (*n* = 124)							
3	3	15.67	2.08	0.33	0.58	0.00	0.00
4	17	23.76	6.72	2.82	1.19	0.65	1.37
5	26	26.85	6.28	3.73	0.67	2.27	1.82
6	18	32.28	5.82	4.00	0.00	2.61	1.69
7	12	43.83	5.84	4.00	0.00	3.83	0.58
8	15	50.87	4.79	4.00	0.00	4.00	0.00
9	20	53.05	4.91	4.00	0.00	4.00	0.00
10	13	55.23	2.80	4.00	0.00	4.00	0.00
Children with legal blindness (*n* = 72)							
7	4	26.00	4.55	3.25	0.96	2.25	1.71
8	11	29.18	6.23	3.91	0.30	3.00	1.10
9	9	37.11	6.70	3.89	0.33	3.11	1.17
10	9	41.89	8.07	4.00	0.00	3.56	0.88
11	4	42.00	9.76	4.00	0.00	4.00	0.00
12	16	47.94	9.02	4.00	0.00	4.00	0.00
13	10	50.50	8.52	4.00	0.00	4.00	0.00
14	9	51.67	10.78	4.00	0.00	4.00	0.00
Children with total blindness (*n* = 45)							
7	4	24.25	12.01	1.50	0.58	0.00	0.00
8	4	28.50	3.42	3.50	1.00	1.50	1.92
9	11	35.64	10.07	3.55	1.04	3.00	1.48
10	4	44.00	17.34	3.50	1.00	3.50	1.00
11	5	38.00	8.60	4.00	0.00	4.00	0.00
12	6	51.83	5.15	4.00	0.00	4.00	0.00
13	6	46.83	5.35	4.00	0.00	4.00	0.00
14	5	51.40	6.88	4.00	0.00	4.00	0.00

#### 2.3.2 Difference in False Belief in Children With Legal Blindness and Children With Total Blindness

Table 2 presents the descriptive statistics of children with normal sight, legal blindness, and total blindness aged 7–10 years. For sighted children, first‐ and second‐order false beliefs were not significantly related to other variables (*p* > 0.05). Meanwhile, for children with legal or total blindness, both types of false beliefs were significantly related to verbal ability and age (*p* < 0.05), except for the relationship between age and second‐order false belief among children with legal blindness (*p* > 0.05).

**TABLE 2:** Descriptive Statistics of the main variables of the 7–10‐year‐old children with normal sight, legal blindness, and total blindness.

		*M*	SD	1	2	3	4
Children with normal sight (*n* = 60)	1. First‐order false belief	4.00	0.00	1	—	—	—
	2. Second‐order false belief	3.97	0.26	—	1	0.05	0.20
	3. Verbal ability	51.13	6.08	—	—	1	0.62^**^
	4. Age	8.57	1.05	—	—	—	1
Children with legal blindness (*n* = 33)	1. First‐order false belief	3.85	0.44	1	0.58^**^	0.36^*^	0.38^*^
	2. Second‐order false belief	3.09	1.16	—	1	0.54^**^	0.32
	3. Verbal ability	34.42	8.81	—	—	1	0.67^**^
	4. Age	8.70	1.02	—	—	—	1
Children with total blindness (*n* = 23)	1. First‐order false belief	3.17	1.19	1	0.80^**^	0.63^**^	0.52^*^
	2. Second‐order false belief	2.30	1.80	—	1	0.68^**^	0.68^**^
	3. Verbal ability	33.87	12.28	—	—	1	0.52^*^
	4. Age	8.65	0.98	—	—	—	1

*Note:* The same statistical notations apply to subsequent tables.

Abbreviations: M, mean; SD, standard deviation.

^**^ Significant correlation at the *α* = 0.01 level.

^*^ Significant correlation at the *α* = 0.05 level.

Independent‐samples t tests indicated no significant differences in age (*t*_(54)_ = 0.17, *p* = 0.870, *d* = 0.05), verbal ability (*t*_(54)_ = 0.20, *p* = 0.844, *d* = 0.05), or second‐order false belief (*t*_(34.56)_ = 1.85, *p* = 0.073, *d* = 0.63) between children with legal and total blindness. However, children with legal blindness scored significantly higher than those with total blindness on first‐order false belief (*t*_(26.24)_ = 2.59, *p* < 0.05, *d* = 1.01).

#### 2.3.3 Interaction Effect of Visual Impairment and Verbal Ability on False Belief

We formulated two moderation models to investigate the effect of visual impairment and verbal ability on false belief scores. Visual impairment was treated as the independent variable and operationalized as a dummy variable. Meanwhile, verbal ability served as the moderating variable. Age was included as a control variable. The models used first‐ and second‐order false belief scores as dependent variables. For the dependent variables, the first model explained 51.65% of the variance (*F*_(6,109)_ = 19.41, *p* < 0.001), whereas the second model explained 58.34% of the variance (*F*_(6,109)_ = 25.44, *p* < 0.001).

Table 3 indicates that verbal ability significantly (*p* < 0.01) moderated the difference in the first‐order false belief scores between children with total blindness and children with normal sight. However, verbal ability failed to moderate the difference in first‐order false belief scores between children with legal blindness and children with normal sight (*p* > 0.05).

**TABLE 3:** Interaction effect of visual impairment and verbal ability on first‐order false belief.

Variable	Effect	SE	*t*	*p*	95% LLCI	95% ULCI	*R* ^2^
Constant	0.45	0.17	2.59	0.011	0.11	0.79	51.65%
X1	−0.23	0.27	−0.86	0.392	−0.77	0.30	
X2	−0.61	0.27	−2.27	0.025	−1.14	−0.08	
Verbal ability	−0.18	0.21	−0.87	0.389	−0.60	0.23	
Int1	0.38	0.25	1.52	0.132	−0.12	0.88	
Int2	1.22	0.24	5.04	< 0.001	0.74	1.71	
Age	0.15	0.08	1.72	0.088	−0.02	0.31	

*Note:* All continuous variables are standardized. X1: difference in the impact of Groups 1 and 0 on first‐order false belief; X2: difference in the impact of Groups 2 and 0 on first‐order false belief; Group 0: children with normal sight; Group 1: children with legal blindness; Group 2: children with total blindness; Int1: X1 × Verbal ability; Int2: X2 × Verbal ability.

Table 4 indicates that when verbal ability was low (−1 SD), the first‐order false belief scores of children with total blindness were significantly lower than those of children with normal sight (*p* < 0.01). However, when verbal ability was high (+1 SD), no difference existed between children with total blindness and children with normal sight and between children with legal blindness and children with normal sight (*p* > 0.05).

**TABLE 4:** Simple slope of visual impairment to first‐order false beliefs at different levels of verbal ability.

Verbal ability	Visual impairment	Effect	SE	*t*	*p*	95% LLCI	95% ULCI
−1 SD	X1	−0.61	0.39	−1.59	0.114	−1.38	0.15
	X2	−1.83	0.40	−4.62	< 0.001	−2.62	−1.05
*M*	X1	−0.23	0.27	−0.86	0.392	−0.77	0.30
	X2	−0.61	0.27	−2.27	0.025	−1.14	−0.08
+1 SD	X1	0.15	0.35	0.42	0.677	−0.55	0.85
	X2	0.62	0.32	1.91	0.058	−0.02	1.26

*Note:* All continuous variables are standardized. X1: difference in the impact of Groups 1 and 0 on first‐order false belief; X2: difference in the impact of Groups 2 and 0 on first‐order false belief; Group 0: children with normal sight; Group 1: children with legal blindness; Group 2: children with total blindness; Int1: X1 × Verbal ability; Int2: X2 × Verbal ability.

Table 5 shows that the differences in second‐order false belief scores between children with total blindness and children with normal sight and between children with legal blindness and children with normal sight were moderated by verbal ability (*p* < 0.01).

**TABLE 5:** Interaction effects of visual impairment and verbal ability on second‐order false belief.

Variable	Effect	SE	*t*	*p*	95% LLCI	95% ULCI	*R* ^2^
Constant	0.61	0.16	3.77	0.000	0.29	0.93	58.34%
X1	−0.46	0.25	−1.84	0.069	−0.96	0.04	
X2	−0.82	0.25	−3.31	0.001	−1.31	−0.33	
Verbal ability	−0.17	0.20	−0.89	0.374	−0.56	0.21	
Int1	0.74	0.23	3.16	0.002	0.27	1.20	
Int2	1.08	0.23	4.77	< 0.001	0.63	1.52	
Age	0.16	0.08	1.98	0.051	0.00	0.31	

*Note:* All continuous variables are standardized. X1: difference in the impact of Groups 1 and 0 on second‐order false belief; X2: difference in the impact of Groups 2 and 0 on second‐order false belief; Group 0: children with normal sight; Group 1: children with legal blindness; Group 2: children with total blindness; Int1: X1 × Verbal ability; Int2: X2 × Verbal ability.

Table 6 shows that children with total blindness and children with legal blindness recorded significantly lower second‐order false belief scores than those with normal sight (*p* < 0.01) when verbal ability was low (−1 SD). The second‐order false belief scores of children with legal blindness were equal to those of children with normal sight when verbal ability was moderate (at the mean level; *p* > 0.05), whereas no significant difference existed between children with total blindness and children with normal sight (*p* > 0.05) when verbal ability was high (+1 SD).

**TABLE 6:** Simple slope of visual impairment to second‐order false belief at different levels of verbal ability.

Verbal ability	Visual impairment	Effect	SE	*t*	*p*	95% LLCI	95% ULCI
−1 SD	X1	−1.20	0.36	−3.35	0.001	−1.91	−0.49
	X2	−1.90	0.37	−5.16	< 0.001	−2.63	−1.17
*M*	X1	−0.46	0.25	−1.84	0.069	−0.96	0.04
	X2	−0.82	0.25	−3.31	0.001	−1.31	−0.33
+1 SD	X1	0.27	0.33	0.83	0.408	−0.38	0.92
	X2	0.26	0.30	0.85	0.395	−0.34	0.85

*Note:* All continuous variables are standardized. X1: difference in the impact of Groups 1 and 0 on second‐order false belief; X2: difference in the impact of Groups 2 and 0 on second‐order false belief; Group 0: children with normal sight; Group 1: children with legal blindness; Group 2: children with total blindness; Int1: X1 × Verbal ability; Int2: X2 × Verbal ability.

### 2.4 Discussion

By focusing on the first‐ and second‐order false belief scores of children with CVI, Study 1 examines the effects of CVI and verbal ability on ToM development. Based on TEC, we hypothesized that CVI is associated with delayed ToM development with verbal ability playing a moderating role. The results indicate that children with total blindness scored significantly lower on false belief tasks than sighted children, whereas those with legal blindness scored in between. Moreover, verbal ability moderated the relationship between CVI and false belief scores, though patterns differed for children with total versus legal blindness. These findings support the hypothesis and highlight the compensatory role of verbal ability in ToM development in children with CVI.

#### 2.4.1 Delay in ToM Development in Children With CVI

Based on a clear definition of CVI, strict inclusion criteria, and a well‐defined age range, Study 1 found that CVI was associated with delayed ToM development. The severity of CVI determined the degree to which ToM development was delayed. Specifically, the more severe the CVI, the greater the negative effect on ToM development. The performance of children with total blindness was significantly lower than that of children with normal sight, with the performance of children with legal blindness falling in between. The findings are consistent with studies on perspective taking in children with blindness (Miletic 1995).

Previous studies on first‐order false beliefs have demonstrated that ToM development in children with CVI is delayed, with visual impairment occurring prior to the sensitive period for first‐order false belief (Green et al. 2004; Michael et al., 2010; Minter et al. 1998; Roch‐Levecq 2006). In these studies, ToM development in children with CVI remained delayed even when language was matched. The results of this study align with these studies. In Study 1, children with total blindness experienced more difficulties in understanding false belief compared with children with legal blindness of similar age and verbal ability. Green et al. (2004) also argued that the absence of vision could result in disruptions in ToM development.

Based on TEC, several explanations may account for the effect of CVI on ToM. First, CVI influences the understanding of causal links between expressions and social context. Children with CVI often fail to infer relationships between others’ facial expressions and events due to the lack of joint visual attention (Roch‐Levecq 2006). For example, Brandone and Stout (2023) found a significant longitudinal association between joint attention and ToM. Second, CVI affects observation of others’ behavior. Imbiriba et al. (2013) argued that early visual impairment influences motor representation of body movements, and understanding behavior is essential for understanding mental states (Ruffman 2023).

Third, CVI affects self‐experience, shaping the understanding of others' mental states. Bedny and Saxe (2012) showed that blind individuals' sensory representation is altered, leading to distorted body schema (Schott et al. 2021) and affecting their self‐experience. From a neuroscientific perspective, the temporoparietal junction (TPJ) is linked to sensory integration (Kerr 2008) and ToM (Kerr 2008; Schurz et al. 2014). CVI may thus affect ToM by altering TPJ functioning. For example, research on perspective taking indicates that adults with blindness show different LPP latencies in the right TPJ compared with sighted adults (Papageorgiou et al. 2020). Thus, the self‐forms the foundation for understanding others (Bradford et al. 2018).

Finally, CVI may foster a body‐centered reference pattern. Roch‐Levecq (2006) suggested that individuals with blindness rely on their bodies as the primary reference in social interactions, potentially hindering perspective taking. There is evidence that children with visual impairment perform worse on perspective‐taking tasks than sighted children (Miletic 1995). As perspective taking is central to ToM, this body‐centered reference may impair mental rotation abilities by impeding the coordination and suppression of self–other perspectives, which are necessary processes for ToM development.

Our findings diverge from those of two earlier studies (Bartoli et al. 2019; Pijnacker et al. 2012), which reported that congenitally blind children performed comparably to sighted children in false belief tasks. However, these studies did not clearly define CVI, even though verbal ability was matched. Michael et al. (2010), Green et al. (2004), Minter et al. (1998), and Roch‐Levecq (2006) clearly defined CVI (at birth or before 1 year of life) and observed a delay in ToM development in children with CVI. Likewise, in this study, children with total blindness performed poorly in the false belief task. Furthermore, the previous studies recruited more children with low vision than children with total blindness. The current study and Roch‐Levecq (2006) demonstrated a correlation between residual vision and ToM, with children with low vision performing better on perspective‐taking tasks than children with total blindness (Miletic 1995). Accordingly, residual vision and unclear definitions of CVI may explain the differences between the findings. Specifically, an unclear definition of CVI may indicate that the onset of visual impairment occurred after the ToM‐sensitive period (3 years old). Visual experiences during infancy contribute to ToM development. Ruffman (2023) argued that visual experiences help infants predict behavior and acquire mental state words; afterward, infants began to understand mental states. Visual impairment prevents children with blindness from witnessing the actions and reactions of other people, leading to differences in experiences compared with sighted children (Bedny and Saxe 2012). Sharing similar experiences can help in understanding others. In other words, blind children whose visual impairment occurs after the ToM‐sensitive period can witness behavior, undergo visual experiences similar to those of sighted children, and learn mental state words. These visual experiences promote ToM development.

Vision is crucial to ToM development in early childhood. Longitudinal studies could be conducted to further examine the dynamic interplay between visual experience and ToM over time. By tracking children with varying degrees of visual impairment from infancy through early childhood, such an approach could clarify how early visual input shapes ToM. According to TEC, hearing and other senses may also contribute to such differences; therefore, future studies should target these as areas for investigation and intervention.

#### 2.4.2 Moderating Role of Verbal Ability

Consistent with previous studies (Bartoli et al. 2019; Green et al. 2004; Pijnacker et al. 2012), verbal ability in children with legal and total blindness was significantly correlated with ToM. Verbal ability in children with CVI moderated ToM development: the negative effect of CVI on ToM diminished as language scores increased, with children with legal blindness showing improvement earlier than those with total blindness. This finding aligns with those of Bäckman and Dixon (1992), who argued that the severity and onset of impairment and task type influence the likelihood of successful compensation for sensory deficits. Moreover, audition and other senses produce a compensatory effect when visual impairment adversely affects perspective taking (Papageorgiou et al. 2020). Likewise, Sorokowska et al. (2018) found a compensatory effect of blindness on auditory perception when blind people assessed others. Older people also rely on superior verbal ability in ToM tasks (Mahy et al. 2014).

There may be two reasons underlying the compensatory role of verbal ability. First, language is a factor in ToM development; for children with CVI, good verbal ability enables them to acquire comprehension and reasoning abilities. Second, the advantage in verbal working memory reported by children with CVI may explain the compensatory effect. Compared with sighted children, an advantageous verbal working memory enables children with CVI to acquire more cognitive resources. Presenting all ToM stories in an auditory format is not beneficial for sighted children, who require more working memory capacity to process such stories (Bartoli et al. 2019; Pijnacker et al. 2012). Sighted children perform poorly in processing auditory information (Pijnacker et al. 2012) compared with children with blindness, and Arcos et al. (2022) reported superior performances among people with blindness in memory for auditory verbal information compared with controls.

#### 2.4.3 Implications

The results of Study 1 support our hypothesis. First, CVI is associated with delayed ToM development. According to TEC, sensory experience underpins ToM; visual impairment limits children's observation and interpretation of others' mental states, thereby impeding ToM development. Second, verbal ability moderates this association. As the children's verbal ability improved, the gap between children with CVI and sighted peers narrowed.

These findings provide significant practical implications. Residual vision and other non‐visual senses should be maximized for children with CVI, particularly in the pre‐linguistic stages of ToM. Studies have shown that audition and other senses compensate in social assessment and perspective taking (Papageorgiou et al. 2020; Sorokowska et al. 2018). In addition, verbal ability should be emphasized to narrow differences between children with CVI and their sighted peers. In conclusion, CVI is linked to delays in ToM development, but verbal ability plays a moderating role and constitutes a key target for intervention.

## 3 Study 2

In Study 1, children with CVI aged 11 fully passed first‐ and second‐order false belief tasks. The saturation of these tasks limited exploration of ToM in older children. To address this gap and broaden the study's age range and scope, Study 2 investigated ToM development in adolescents with CVI.

### 3.1 Method

#### 3.1.1 Participants

To test the second hypothesis, we constructed a regression model. Following previous studies linking residual vision and ToM (Roch‐Levecq 2006) and verbal ability and ToM (Milligan et al. 2007), we assumed a medium effect size of *f* ^2^ = 0.15 (Cohen 1988). With a statistical power of 1 − *β* = 0.8 and *α* = 0.05, the sample size was estimated at approximately 77 participants using the pwr package in R. The inclusion criteria were the same as those of Study 1. All participants in Study 1 were included, and each of them completed the verbal ability test only once. The final sample comprised 166 children and adolescents with CVI (aged 7–19; 103 boys; mean age: 12.5 years; SD: 3.38) from five schools.

#### 3.1.2 Procedure

The procedure was similar to Study 1. Participants received compensation for their time and effort after completing Study 2.

### 3.2 Measures

#### 3.2.1 Residual Vision

School doctors examined and reported residual vision, with a mean of 0.02 (SD 0.038). Following Roch‐Levecq (2006), these scores were converted to the logarithm of the minimum angle of resolution (logMAR), a widely used clinical measure of visual acuity, yielding an average of 1.98 (SD: 0.48).

#### 3.2.2 Theory of Mind

The Faux pas recognition test (Baron‐Cohen et al. 1999; Fafsca et al. 2016) was administered, including 10 faux pas and 10 control stories, with scores calculated separately. For faux pas stories, correct responses to six follow‐up questions were scored as 1, yielding a maximum of 60. For control stories, correct responses to the first question were scored as 2, yielding a maximum of 20. Following Fafsca et al. (2016), specific story scores were subtracted from the total if the corresponding control response was incorrect.

#### 3.2.3 Verbal Ability

As in Study 1, children completed the vocabulary subscale of the Wechsler Intelligence Scale for children.

#### 3.2.4 Statistical Analysis

Descriptive, correlational, and regression analyses were conducted in SPSS to identify the relationships among age, verbal ability, residual vision, and ToM. To enhance comparability across differently scaled variables, all variables were standardized using z‐score transformation.

### 3.3 Results

Table 7 shows that residual vision was significantly negatively correlated with verbal ability (*p* < 0.01), age (*p* < 0.01), and faux pas recognition (*p* < 0.05). Meanwhile, verbal ability, age, and faux pas recognition were significantly positively correlated (*p* < 0.01).

**TABLE 7:** Descriptive statistics and correlation analysis of key variables.

	*M*	SD	1	2	3	4
1. Residual vision (logMAR)	1.98	0.48	1	−0.21^**^	−0.31^**^	−0.15^*^
2. Verbal ability	45.99	12.42	—	1	0.77^**^	0.79^**^
3. Age	12.50	3.38	—	—	1	0.74^**^
4. Recognition of faux pas	43.52	17.55	—	—	—	1

^**^ Significant correlation at the *α* = 0.01 level.

^*^ Significant correlation at the *α* = 0.05 level.

Using age, residual vision (logMAR), and verbal ability as independent variables and faux pas scores as the dependent variable, the regression model explained 66.7% of variance (*F*_(3,162)_ = 108.19, *p* < 0.001). As shown in Table 8, age and verbal ability exerted significant positive effects (*p* < 0.01), whereas residual vision was not significant (*p* > 0.05).

**TABLE 8:** Regression analysis of age, residual vision, and verbal ability on faux pas recognition.

	*B*	SE	*t*	*p*	95% LLCI	95% ULCI	*R* ^2^
Age	0.36	0.07	4.99	< 0.001	0.22	0.51	66.70%
Residual vision (logMAR)	0.07	0.05	1.47	0.144	−0.02	0.16	
Verbal ability	0.52	0.07	7.37	< 0.001	0.38	0.66	

*Note:* Dependent variable: recognition of faux pas. All continuous variables are standardized.

### 3.4 Discussion

By employing the faux pas recognition test and extending the age range to adolescence, Study 2 examined how age, verbal ability, and residual vision jointly shape ToM development in CVI. Following Bedny and Saxe's (2012) proposal that ToM emerges through prelinguistic and linguistic phases, we hypothesized positive effects of age and verbal ability but not of residual vision. The regression results confirmed these predictions, reinforcing verbal ability as a key target for intervention.

Age strongly predicted ToM in children with CVI, consistent with previous studies (Bigelow et al. 2021; Mahy et al. 2014; Peterson et al. 2000). ToM develops with age (Meinhardt‐Injac et al. 2020; Osterhaus and Koerber 2021), and Meinhardt‐Injac et al. (2020) emphasized its link to social interactions and maturation of specific brain regions. Thus, even without typical visual input, the age‐related ToM development observed in children with CVI may reflect the accumulation of social experiences and the time required for the ToM brain network to mature. With age, children and adolescents with CVI gain increased opportunities for social interactions, and their ToM becomes associated with social competence, prosocial behavior, and peer popularity (Slaughter et al. 2015). These developments are supported by key brain regions such as the TPJ and medial prefrontal cortex (Schurz et al. 2014). Notably, Bedny et al. (2009) found that ToM brain regions in sighted and adults with congenital blindness were similarly localized and functionally specific.

The significant predictive role of verbal ability aligned with previous findings. For example, Meinhardt‐Injac et al. (2020) showed that ToM development in participants aged 11–25 corresponds to improvements in language ability. Verbal ability mediates the relationship between age and ToM throughout early and middle childhood (Bigelow et al. 2021), which underlines the majority of the effect of socioeconomic status on adolescents' ToM ability (Pluck et al., 2021). Moreover, longitudinal studies have confirmed that verbal ability development predicts ToM (Osterhaus and Koerber 2021; Siu and Cheung 2022). While language promotes cognitive development, the link between language and cognition transcends initial cognition (Perszyk and Waxman 2018).

In Study 2, residual vision was correlated with ToM but was not a predictor after controlling for age and verbal ability, echoing the results of Roch‐Levecq (2006). The results of Study 2 showed that visual information was not the sole determining factor of ToM in youth with CVI. Similarly, Bedny and Saxe (2012) suggested that the process of understanding other minds progresses through the pre‐linguistic and linguistic phases. The pre‐linguistic phase is driven by visual experience and direct observation of others' actions, whereas the linguistic phase is dominated by verbal input and learning about mental states through communication. Meanwhile, Brandone and Stout (2023) conducted a longitudinal study, finding that the joint visual attention of infants predicts later ToM. Based on this evidence, the changing roles of vision and verbal ability in ToM development may explain the findings.

In the early stages of human development, especially the pre‐linguistic phase, vision plays a dominant role in ToM development because it helps infants observe others' behaviors and learn mental state words, promoting the link between language and cognition. As verbal ability develops and improves, the role of vision gradually diminishes, whereas that of verbal ability gradually increases. In summary, verbal ability exerts a more long‐lasting and profound impact on ToM development compared with vision.

The results of Study 2 support our hypothesis. Age and verbal ability significantly predict ToM development in youth with CVI, whereas residual vision does not. These findings align with those of Bedny and Saxe (2012). Notably, in the pre‐linguistic phase, vision plays a crucial role in ToM, but as children age, verbal ability becomes increasingly important.

These findings have notable practical implications. First, the extended developmental period of ToM provides sufficient time for intervention and research. Second, emphasis should be placed on strengthening verbal ability in youth with CVI to support ToM development.

## 4 General Discussion

This study aimed to explore the roles of CVI, verbal ability, and residual vision in ToM development in children and adolescents with CVI. To our knowledge, this is the first study to employ two paradigms. In Study 1, the findings were consistent with most existing research, showing delays in ToM among children with CVI. Verbal ability moderated these delays, with children with total blindness performing worse than those with legal blindness, even when matched on age and verbal ability. Meanwhile, in Study 2, age and verbal ability significantly predicted ToM development, whereas residual vision did not. Thus, although CVI impeded ToM development at the earliest stages, age and verbal ability became stronger predictors over time. Drawing on TEC, we examined mechanisms by which CVI affects ToM development. Overall, the findings show how age and verbal ability offset the ToM delays associated with CVI, offering guidance for targeted interventions. The protracted trajectory of ToM also affords an extended window for such interventions.

### 4.1 Implications

The findings of this study advance our theoretical understanding of ToM development in children and adolescents with CVI and the design of practical interventions. Grounded in TEC, the study clarifies the mechanisms by which ToM develops in this population, emphasizing the dynamic interplay of sensory input, verbal ability, brain maturation, and social experience. This approach extends TEC's application in CVI and provides a new lens through which to examine the complexity of cognitive development. Practically, the findings provide systematic guidance for interventions at the individual, family, educational, and societal levels:

Individual level. Children with CVI should maximize their residual vision and non‐visual senses to interpret others' actions, emotions, and intentions. Given the pivotal role of language in ToM development, strengthening verbal skills is essential for decoding others' emotions and intentions. Activities such as engaging with traditional Chinese culture and reading literary works may further foster ToM (Chen and Xu 2020; Han and Kong 2025). Spiritual practices may also help integrate the four‐fold self, which provides the foundation for understanding others. Although congenital visual impairment affects the egocentric and sociocentric self, spirituality can support self‐understanding and self‐integration (Han and Kong 2025; Wang et al. 2020).

Family level. Parents should regularly use mental‐state terms in daily conversations, integrate multisensory cues, and establish secure attachment relationships. Familiarity with their child's developmental profile and the use of evidence‐based strategies can further strengthen ToM development.

Educational level. Researchers should design stage‐specific intervention programs aligned with children's age and level of cognitive development. Interdisciplinary teams should collaborate to create integrated intervention systems that coordinate sensory, linguistic, familial, and social resources to address diverse needs.

Societal level. Communities can foster inclusion by organizing events and volunteer opportunities that expand social networks and enhance social confidence, fostering an accessible and friendly community environment. Governments should promote equity in resource allocation and strengthen support systems to reduce social isolation and enable full participation of individuals with CVI.

#### 4.1.1 Limitations and Future Research Directions

This study has some limitations. First, the sample excluded children aged zero to six years, adults, and individuals with acquired visual impairment. Recruitment challenges (Begeer et al. 2014) limited coverage of these groups, restricting our ability to assess vision's role across the full developmental spectrum. Specifically, the absence of infants (0–12 months; primary sensory integration stage) and toddlers (12–36 months; emergent symbolic representation stage) prevented examination of how visual deprivation affects the earliest stages of the developmental cascade: low‐level social perception → intention understanding → high‐level ToM. This omission risks underestimating the importance of visual input in the prelinguistic phase.

Second, the study did not examine other determinants, such as executive function, joint attention, imitation, or family environment that pose challenges for individuals with blindness but remain underexplored. Future studies should systematically address these factors. Finally, longitudinal and neuroimaging studies should be conducted to explore life‐span patterns and the neural mechanisms of ToM development. Longitudinal designs would help identify critical periods for language and ToM, enabling targeted interventions to promote ToM effectively.

## 5 Conclusion

This study is the first to adopt two research paradigms and establish clear participant inclusion criteria to demonstrate the role of CVI, verbal ability, and age in ToM development among children and adolescents with CVI. Study 1 showed that CVI was associated with a delay in ToM development, and language played a moderating role. By expanding the age range to 19 years, Study 2 further revealed that age and language predicted ToM development, whereas residual vision did not have a significant effect. CVI limits access to mental‐state cues, thereby hindering ToM, whereas language—through verbal scaffolding—can compensate for this deficit. Thus, linguistic input assumes an increasingly compensatory role as verbal ability increases. These findings underscore the need to leverage residual vision and multisensory input and highlight the intervention potential of language‐rich environments within family, school, community, and individual contexts. The protracted ToM development period affords an extended window for intervention, proving to be an advantage for tailored interventions.

## Ethics Statement

This research complies with the Declaration of Helsinki (2023) and is approved by the Institutional Review Board of the Institute of Psychology, Chinese Academy of Sciences.

## Conflicts of Interest

All authors declare no conflicts of interest.
